# Determination of strongly overlapping signaling activity from microarray data

**DOI:** 10.1186/1471-2105-7-99

**Published:** 2006-02-28

**Authors:** Ghislain Bidaut, Karsten Suhre, Jean-Michel Claverie, Michael F Ochs

**Affiliations:** 1 Fox Chase Cancer Center, 333 Cottman Avenue, Philadelphia, PA, 19111, USA; 2 Structural and Genomic Information Laboratory, UPR2589-CNRS, 13288 Marseille, France; 3 Center for Bioinformatics, Department of Genetics, University of Pennsylvania School of Medicine, 1423 Blockley Hall, 423 Guardian Drive, Philadelphia, PA 19104-6021, USA

## Abstract

**Background:**

As numerous diseases involve errors in signal transduction, modern therapeutics often target proteins involved in cellular signaling. Interpretation of the activity of signaling pathways during disease development or therapeutic intervention would assist in drug development, design of therapy, and target identification. Microarrays provide a global measure of cellular response, however linking these responses to signaling pathways requires an analytic approach tuned to the underlying biology. An ongoing issue in pattern recognition in microarrays has been how to determine the number of patterns (or clusters) to use for data interpretation, and this is a critical issue as measures of statistical significance in gene ontology or pathways rely on proper separation of genes into groups.

**Results:**

Here we introduce a method relying on gene annotation coupled to decompositional analysis of global gene expression data that allows us to estimate specific activity on strongly coupled signaling pathways and, in some cases, activity of specific signaling proteins. We demonstrate the technique using the Rosetta yeast deletion mutant data set, decompositional analysis by Bayesian Decomposition, and annotation analysis using ClutrFree. We determined from measurements of gene persistence in patterns across multiple potential dimensionalities that 15 basis vectors provides the correct dimensionality for interpreting the data. Using gene ontology and data on gene regulation in the Saccharomyces Genome Database, we identified the transcriptional signatures of several cellular processes in yeast, including cell wall creation, ribosomal disruption, chemical blocking of protein synthesis, and, criticially, individual signatures of the strongly coupled mating and filamentation pathways.

**Conclusion:**

This works demonstrates that microarray data can provide downstream indicators of pathway activity either through use of gene ontology or transcription factor databases. This can be used to investigate the specificity and success of targeted therapeutics as well as to elucidate signaling activity in normal and disease processes.

## Background

Many diseases develop because of errors in signaling, and newer therapeutics specifically target proteins involved in cellular signaling [1,2]. However, these therapies are not always effective [3], and the reason for failure, whether inherent poor interaction or complex cellular response, is unknown. In order to understand the development of disease and drug resistance in these cases, the recovery of the process that led to the specific cellular malfunction must be identified. Such errors generally involve the cellular signaling networks that control cell growth, differentiation, apoptosis, and motility [4,5]. Because of the extreme underlying biological complexity of these pathways, diseases that involve errors in signaling processes arise from a myriad of different cellular malfunctions, for example in cancers [6,7] and diabetes [8,9]. It is from this complex background that functional genomics attempts to glean insight to improve our understanding of diseases.

One of the major uses of microarrays has been elucidation of gene expression in cancer, often focused on refining cancer identification using computational and statistical approaches [10-12]. In addition, the discovery of biomarkers in the form of differential levels of production of mRNA has been a focus in a number of studies [13-15]. The fact that determination of the mRNA levels of a single gene is easier than using an entire array has driven the shift to the use of arrays to generate potential biomarkers, so that the expression levels of these individual genes can be screened for in a more economical way (see, for example, [16]). For diabetes, microarrays have been used to elucidate gene expression in both type I and type II diseases, and customized chips targeting genes of interest have been developed [17].

Many tools for statistical inference, pattern recognition, and data mining have been developed for microarray data analysis. Statistical tests include SAM [18], VERAandSAM [19], ANOVA techniques [20,21], Bayesian approaches [22,23], and rank tests [24]. Pattern recognition and data mining techniques comprise both unsupervised techniques, such as hierarchical clustering [25], singular value decomposition [26], multidimensional scaling [27], Bayesian mixture models [28,29], and other clustering methods [30-34], and supervised techniques, such as support vector machines [35] and artificial neural networks [36], (for a review see [37]).

While these techniques are useful, they have certain limitations as regards more advanced uses in the elucidation of mechanisms operating in diseased tissues. New therapeutics specifically target proteins involved in cellular signaling [1-3,38-40]. As noted above, these therapies are not always effective, and a method to understand the reason for their ineffectiveness is highly desirable. If the failure modes for the targeted therapeutics are understood, new therapeutics can be designed or combination therapies undertaken. In addition, to design new therapies that work alone or in combination with other therapies, an understanding of signaling networks is required. Microarray measurements can provide insight into these issues.

Unfortunately, the recovery of pathway information from transcriptional data requires complex analysis, since signaling protein activity is not generally linked to the mRNA expression levels of genes encoding the signaling proteins themselves [41], nor are protein levels tightly coupled to transcript levels even in yeast [42,43]. This makes it impossible to directly link an increase in mRNA expression of the gene encoding a signaling protein, such as the therapeutic target, with activity of the protein and therefore of the signaling pathway. Instead, an analysis must treat changes in mRNA levels as downstream indicators of activity.

An important issue to resolve in order to correctly interpret patterns in microarray data is the underlying dimensionality of the data, since statistical analysis of genes in groups relies on correct separation. The dimensionality provides an estimate of the number of patterns required to explain the variation in the data not related to noise, which is equivalent to the number of basis vectors required mathematically to describe the data or the number of principal components required to span the data.

**Figure 1:**
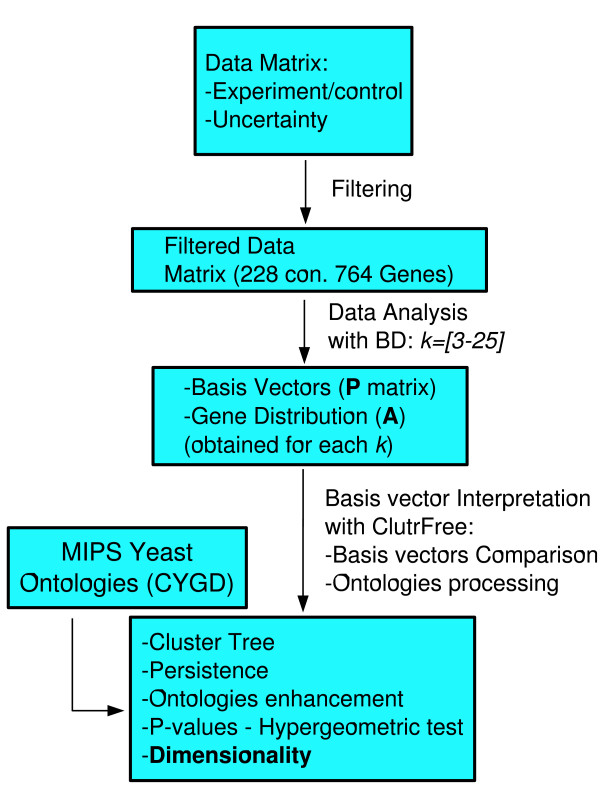
**Data analysis flowchart**. The data was downloaded from Rosetta Inpharmatics and filtered to include only genes and experiments that showed significant variation. Bayesian Decomposition analysis generated patterns and associated gene lists for all dimensionalities between 3 and 25. ClutrFree was used to interpret these results, including use of the MIPS database of ontologies.

We present here a new application of Bayesian Decomposition [44-48] and ClutrFree [49] that estimates dimensionality by measuring the consistency of assignment of genes to patterns. With this approach, transcriptional signatures are linked to signaling activities through gene ontology [50] using the MIPS database [51] and through analysis of transcription factor activity [52]. We demonstrate this technique on the Rosetta deletion mutant dataset [53], which is a compendium of genome-wide transcription measured for 6300 genes across 300 conditions (mostly deletion mutants, but some chemical treatments). Figure 1 details the workflow of our analysis. Previous studies of the compendium were performed using hierarchical clustering [53], non-negative matrix factorization [54], and Bayesian Decomposition [44]. The dimensionality of the data was estimated in various ways in these studies leading to estimates from 7 to 50 dimensions.

## Results

### Dimensionality of the data

**Figure 2:**
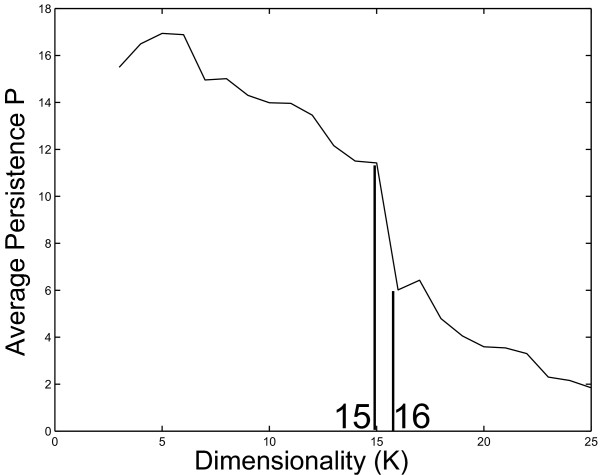
**The average persistence across all dimensions**. The average persistence across the dimensions is plotted for 3 to 25 dimensions. The significant drop between 15 and 16 dimensions suggests that 15 patterns provides the correct dimensionality for analysis.

We propose a value for the Rosetta dataset dimensionality based on the average persistence calculations at each tree level made with ClutrFree using multiple Bayesian Decomposition simulations. The dimensionality has been inferred from the average persistence defined in the Methods section. As the number of basis vectors (i.e., patterns) *k *is increased, the curve shows a dramatic drop for *k *> *15 *(see Figure 2). This drop is due to the reorganization of the groups of mutants constituting the basis vectors for *k *> *15 *patterns, leading to an overly low average persistence.

The freedom to move between branches leads also to some loss of consistency in the annotations as one moves down a branch. This contrasts with the behavior of basis vectors obtained for less than *15 *patterns where biological functions split logically as the number of patterns increases. We also observed a reduction in the number of genes related to each basis vector for *16 *patterns in comparison to *15*. Also, the standard deviation across samples of the obtained vectors is significantly higher for *16 *patterns (1.7 × 10^-3^) than for 15 patterns (7.9 × l0^-4^) indicating that the Markov chain sampling is not as tightly constrained by the probability distribution. The behavior observed occurs because of the potential to overfit the data with *16 *basis vectors allowing the algorithm to find multiple configurations to explain the variation in the data.

### Identifying patterns and functions

Bayesian Decomposition retrieves the two linked matrices: the **P **matrix (pattern matrix) groups mutants that share cellular functions, which can be deduced from the genes linked to each pattern contained in the **A **matrix. Each mutant (a column of the **P **matrix) can belong to multiple patterns, which models the fact that each mutant will have many cellular functions active. Each gene (a row of the **A **matrix) can be assigned to multiple patterns, reflecting the fact that evolution has led to genes being involved in multiple cellular processes. Interpretation of the results involves identifying cellular processes from the genes that are significantly expressed in a pattern (i.e., within a column of **A**).

We proceed by using the dimensionality estimate of *15 *patterns and exploring for each pattern the genes associated with that pattern. These genes are interpreted using the MIPS ontology for yeast [51] in order to predict the cellular processes associated with a pattern. In addition, for patterns that can be linked to signaling pathways, we discuss the use of data on genes regulated by specific transcription factors and validate the results by analysis of specific key deletion mutants. For each pattern that shows enhancement of ontological terms we provide the terms, the enhancement (as defined in the methods section), and the *p *value for a hypergeometric test on the term.

We summarize the results in terms of patterns previously identified in other studies using this data set, then we present the new results isolating signatures for activity of the mating and filametation pathways.

### Patterns identified in previous studies

Examination of pattern 1 shows expression of the overall common minimal processes necessary for survival, with 386 annotated genes associated with this pattern at a 3*σ *level. Measure of enhancement, *e*, of cellular functions, reveals two highly represented functional groups: 1) groups related to protein synthesis and 2) groups related to DNA synthesis. Group 1 includes genes enhanced in Protein Targeting, Sorting and Translocation (Term 14.04, *e *= *1.84*, *p *= 0.0022), Protein Synthesis (Term 12, *e *= 1.55, *p *= 0.024), and Ribosome Biogenesis (Term 12.01, *e *= 1.60, *p *= 0.059). Group 2 includes DNA Processing (Term 10.01, *e *= 1.32, p = 0.097), DNA Recombination and DNA Repair (Term 10.01.05, *e *= 1.31, p = 0.16), and DNA Synthesis (Term 10.01.03, *e *= 1.58, p = 0.16). The *p*-values for the ontology terms remain high, due to the large number of genes associated with this pattern.

This pattern, which essentially includes genes necessary for viability, contains all the mutants of the dataset, although the Ssn6Δ mutant shows a lower level for this pattern than other mutants. As the Ssn6Δ mutant exhibits substantially greater overall expression than any other mutant (including the Tup1Δ mutant with the second highest level), this may reflect the high association of the Ssn6Δ mutant seen in almost all patterns, which will have some gene overlap with this pattern.

Pattern 5 contains 172 annotated genes. Highly enhanced ontologies include transport-related functions: Transported Compounds (Term 20.01, *e *= 2.02, *p *< 10^-4^), C-compound and Carbohydrate Transport (Term 20.01.03, *e *= 2.60, *p *< 3 × 10^-4^), and Cellular Transport, Transport Facilitation and Cellular Routes (Term 20, *e *= 1.62, *p *< 6 × 10^-4^), in addition to other transport terms at *p *< 10^-3^. The pattern contains the two deletion mutants, Ssn6Δ and Tup1Δ, and represents the strong response seen in the original study [53]. Ssn6p and Tup1p form a system of transcriptional repression that appears to be highly conserved in eukaryotes [55]. In yeast, the complex acts as a global transcriptional repressor over a large number of genes (more that 150), coordinating several cellular systems, including haploid specific genes, glucose repressible genes, and oxygen utilization genes [56]. Turning off this repression leads to a large overall increase in gene expression (the overall expression in these two mutants is many fold higher than in other mutants).

Pattern 7 is related to the lack of cell wall functions (Cell Wall, Term 42.01, *e *= 0.0), as 28 cell wall genes (of 32 total) are absent from this pattern, while the other four genes have multiple annotations suggesting they have roles unrelated to Cell Wall function. Enhancement is present for Protein Modification (Term 14.07, *e *= 4.7, *p *< 10^-4^) and Fermentation (Term 02.16, *e *= 4.5, *p *< 0.01). This pattern contains the mutants Gas1Δ and Fks1Δ, which impair cell-wall synthesis, as well as the mutant YER083cΔ, annotated as disrupting the cell wall in the original study [53]. The pattern contains other mutants disrupting ergosterol biosynthesis as well, including Erg2Δ, She4Δ, as well as YER044cΔ. In addition, the pattern includes yeast treated with the drugs that are known to disrupt the cell wall, such as Tunicamycin and Glucosamine.

Pattern 11 is related to ribosomal function, with enhancements in terms for Ribosome Biogenesis (Term 12.01, *e *= 6.32, *p *< 4 × 10^-4^) and Protein Synthesis (Term 12, *e *= 3,89, *p *< 6 × 10^-3^). The pattern contains 8 mutants related to ribosomal proteins, Rpll2aΔ, Rpl27aΔ, Rpl34aΔ, Rpl6bΔ, Rp18aΔ, Rps24aΔ, Rps24aΔ (haploid), and Rps27bΔ, as well as some mutants with deleted ORFs of unknown function, YOR078wΔ, YMR269wΔ, and YHR034cΔ, proposed to be involved in ribosomal functions [53].

### Patterns related to cellular signalling pathways

**Figure 3:**
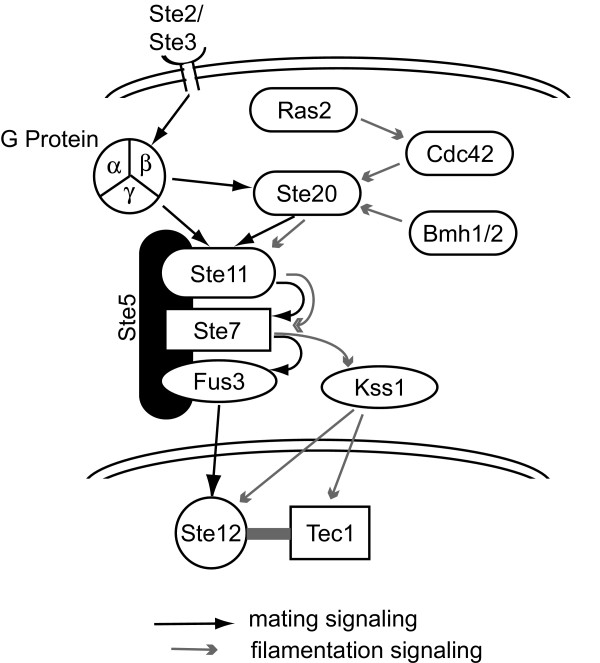
**Yeast MAPK signaling for mating and filamentation**. The strongly linked MAPK signaling pathways for mating and filamentation are shown schematically with black arrows indicating mating pathway signaling and gray arrows showing filamentation pathway signaling. The mating pathway is initiated by binding to Ste2p or Ste3p receptors, while the causative molecular trigger for filamentation is unclear. The pathways share many components.

The two patterns that represent new insights into this data are 13 and 15, which appear related to two strongly coupled developmental pathways in yeast. Previous studies [44,53] have identified the mating pathway transcriptional response, however this has included both the filamentation response and the mating response. It is difficult to separate these signatures, as the mating and filamentation pathways share many common elements in a MAPK cascade as shown in Figure 3[57-59].

Gene ontology (GO) was used to determine the biological function described by each pattern, with a term added specifically for transposable elements, as these are known to play a role during filamentation [60,61]. The terms that showed enhancement are summarized in Table 1. The patterns show strong overlap, since many genes are shared between the mating and filamentation responses. However, the filamentation ontology term is significantly higher only in pattern 15, which also shows a strong signature of transposable element genes. Meanwhile, the GO terms for meiosis and morphogenesis (such as for budding in *S. cerevisiae*) are significantly enhanced only in pattern 13. This allows association of pattern 13 with activation of the mating pathway, and pattern 15 with activation of the filamentation pathway.

**Table 1:** The most enhanced gene ontology terms in patterns 13 and 15. Each term is presented together with a measure of how overrepresented it is compared to a random draw of the same number of genes. These were also confirmed to be significant by hypergeometric tests.

**Pattern 13**		**Pattern 15**	
Pheromone response, mating-type determination	6.31	Transposable elements, viral and plasmid	8.42
development	6.09	Pheromone response, mating-type determination	7.26
Fungal/microorganism development	6.09	Transmembrane signal transduction	6.41
Mating (fertilization)	6.09	G-protein mediated signal transduction	6.10
Transmembrane signal transduction	4.98	development	5.69
Chemoperception and response	4.47	Fungal/microorganism development	5.69
Cellular sensing and response	4.25	Mating (fertilization)	5.69
Interaction with cellular environment	2.94	Chemoperception and response	5.47
Meiosis	2.86	Cellular sensing and response	5.21
Cell growth/morphogenesis	2.77	Cellular communications	4.45
Cellular communications	2.77	Enzyme mediated signal transduction	3.81
Development of asco- basidio- or zygospore	2.57	Interaction with cellular environment	3.61
Enzyme mediated signal transduction	2.37	Enzyme activator	3.56
Protein kinase cascades	2.37	Intracellular signaling	3.14
G protein mediated signal transduction	2.37	Budding, cell polarity, filamentation	2.87

In addition, we analyzed the 10 genes whose expression is most strongly linked to each pattern. These are shown in Table 2, which summarizes which genes are known to be regulated by the transcriptional activators related to the mating (Ste12p) and filamentation (Ste12p-Tec1p complex) pathways. The results show that the top 10 genes related to pattern 13 have 9 genes of known function, with 8 related to the mating response, of which five are known to be regulated by Ste12p. For pattern 15, 7 of the top 10 genes are known to be transposable element genes, with three other genes having unknown functions. This again links pattern 13 to mating and pattern 15 to filamentation.

**Table 2:** The genes most strongly associated with patterns 13 and 15 in order of strength of association. For pattern 13, it is noted whether the genes are known to be regulated in the mating process, and whether the gene is known to be directly regulated by Stel2p. For pattern 15, the gene function is shown. All data is from the Saccharomyces Genome Database [73, 74].

**Pattern 13**			**Pattern 15**	
*Gene*	*Mating?*	*Ste12p*	*Gene*	*Function*

Fig1	Yes	No	YCL019W	Transposable element gene
Prm6	Yes	Yes	YER138C	Transposable element gene
Fus1	Yes	Yes	YER117C	Verified ORF, Prm5
Ste2	Yes	Yes	YER160C	Transposable element gene
Aga1	Yes	No	YJR029W	Transposable element gene
Fus3	Yes	Yes	YBR012W	Removed from SGD
Pes4	No	No	YML045W	Transposable element gene
Prm1	Yes	Yes	YAR009C	Transposable element gene
ORF	--	--	YJR027W	Transposable element gene
Bar1	Yes	No	YLR334C	Hypothetical ORF

In order to validate that the patterns were actually measuring activity of the mating and filamentation pathways, we explored deletion mutants related to these pathways [61,62]. The mating response in *S. cerevisiae *is mediated via a MAPK signaling cascade initiated by binding to the Ste2p or Ste3p membrane receptors (Figure 3). The signal is transduced through Ste11p, Ste7p, and Fus3p with Ste5p serving as a scaffolding protein. The signal activates the Ste12p transcription factor, leading to transcription of mating response genes. In addition, the signal is transduced to the MAPK cascade from the membrane receptor by a G protein complex or through the Ste20p protein. Pattern 13 shows near zero signal for the deletion mutants Ste11Δ, Ste7Δ Fus3Δ, Ste12Δ, Ste5Δ, and Ste2Δ, while showing signal for deletion mutants of Ste20Δ and Tec1Δ. This is exactly as expected, with the membrane receptor, all signaling proteins in the cascade, and the transcription factor necessary to generate the transcriptional response related to the mating signal (note that the Ste3Δ mutant is not in the data set). Ste20p is not necessary to raise the mating response, since the G-protein complex can trigger activation of Ste11p directly. For pattern 15, the response is very similar. The signal is near zero for the deletion mutants Ste2Δ, Ste11Δ, Ste7Δ, and Ste12Δ. The Fus3Δ mutant shows a signal for pattern 15, as appropriate, while the Fus3Δ, Kss1Δ double deletion mutant does not. In addition, the Tec1Δ mutant shows no signal for pattern 15, indicating that Tec1p is required for filamentation [61]. Finally, the signal is greatly reduced for the Ste20Δ deletion mutant in pattern 15 relative to pattern 13, which agrees with previous work suggesting that the filamentation pathway is more dependent on Ste20p signaling than is the mating pathway [62].

## Discussion

Microarrays and GeneChips™ have become the tools of choice for the investigation of genome-wide transcription in most biological systems. The resulting data comprises noisy estimates of transcription levels for roughly 6,000 genes in yeast to more than 20,000 genes in typical mammalian studies. Numerous statistical and data mining methods have been applied to this data in order to identify individual genes showing differential expression, to identify patterns related to physiological states, and to identify groups of genes comprising biological processes. These studies generally have not focused on the estimation of cellular signaling from the data, despite the prevalence of cellular signaling in many diseases.

As noted in the introduction, the recovery of signaling pathway information from transcription data requires complex analysis, since protein levels do not correlate well with mRNA levels and signaling protein activity is not generally linked to the mRNA expression levels of genes encoding the signaling proteins themselves. As such, changes in mRNA levels are limited to being a downstream indicator of activity. If a complete model for the transcription of genes, including all known transcriptional regulators and biological processes regulating transcription, was available, the inference of activity would be straightforward. Unfortunately, the network models and even gene annotations are still far from being complete. In addition, the growing evidence supporting the important role of non-coding RNAs in regulation of gene expression (including antisense transcripts and micro-RNAs, see for example [63-65]) further undermines the potential of using mRNA species as markers for proteins and their activities [66].

In order to overcome this incompleteness, we have created the method described here. We couple identification of transcriptional signatures with our Bayesian Decomposition algorithm to a consistency analysis for gene assignment to patterns determined by comparison of different dimensionalities using ClutrFree. This allows the identification of the correct dimensionality to be applied to subsequent ontology and transcription factor analyses. ClutrFree is also used to determine the ontological terms enhanced within each pattern and to obtain a list of genes tied to this pattern, which can then be linked to specific transcription factors. In this way, the biological processes associated with conditions can be identified, and inferences can be made on the activity of specific transcription factors. This then allows inference on the activity of signaling pathways, which cannot be obtained with methods previously applied to microarray data. Overall, the method requires many separate steps, each modeling an aspect of the biological system, in order to make proper inferences on signaling from the data.

In the application to the Compendium data presented here, our analysis was able to extract the common features for a set of mutants that eliminated related pathways. As in previous studies, the global transcriptional repressor complex Ssn6-Tupl has been isolated in a single group. In addition, patterns for cell-wall synthesis, ribosomal function, and the global functions necessary for continued viability of yeast were isolated. In contrast to previous analyses of this data, two pathways related to the MAPK cascade were isolated, one related to mating and the other to filamentation. Once the correct dimensionality was determined, Bayesian Decomposition was able to identify transcriptional signatures unique for each pathway. The assignment was validated by an investigation of the deletion mutants known to adversely affect these pathways.

## Conclusion

Microarray studies have been widespread in biological and medical research, often focusing on identification of genes significantly correlated with various disease states. However, many diseases arise from disruptions in cellular signaling, and in these cases gene expression only provides a downstream indicator of signaling activity. This greatly complicates the analysis. The new approach introduced here recovered signatures allowing us to make validated inferences on strongly overlapping signaling pathways.

The results demonstrate that for *Saccharomyces cerevisiae*, the mating and filamentation pathways can be distinguished from transcriptional signatures determined from analysis of microarray data, despite the intrinsic high noise, confounding transcriptional activity, and tightly coupled nature of the pathways. The next step will be to apply these methods to more complex signaling networks in worms, flies, and mammals.

## Methods

### The Rosetta deletion mutant data set

The Rosetta Compendium comprises 300 conditions, including 276 deletion mutants, 11 tetracycline regulated genes, and 13 drug treatments, in *S. cerevisiae *growing in rich medium [53]. The data were generated from a two color cDNA microarray hybridization assay [67], and transcriptional profiles were measured both with technical replication and biological replication (151 mutants). In parallel with the 300 experiments, 63 controls of wild-type *S. cerevisiae *were grown in identical conditions and compared against each other, permitting creation of a gene-specific error model. The data was downloaded from Rosetta Inpharmatics.

### Data preprocessing

The data was filtered to retain only conditions characterized by at least a variation of 3 fold in a minimum of 2 genes. Then all genes that did not vary by 3-fold in at least 2 conditions were also removed, leading to a data matrix comprising 764 genes and 228 conditions.

The data used by Bayesian Decomposition included both the mean log ratio for each data point and the uncertainty in this measurement determined by the Rosetta error model. Since Bayesian Decomposition as applied here requires positivity [45], the log ratios were converted to ratios and the uncertainties propagated to uncertainties on the ratios. Although analysis of residuals suggested that seven dimensions fit the data [44], the analysis presented here suggests that this is due to overestimation of uncertainty in the data. Bayesian Decomposition is not highly sensitive to minor misestimations of noise however, so that this should not be a problem for this analysis.

### Analysis with Bayesian Decomposition

Bayesian Decomposition (BD) has been applied to multiple types of data: *in vivo *spectroscopic data [68], medical imaging [69], microarray data from single cell organisms [44,45], mammalian model organisms [47], humans [70], and on phylogenomic sequence data [71]. A detailed description of the algorithm [46] and a review of applications [48] have been published.

Briefly, BD models the microarray data, comprising a matrix of estimates of the ratio of expression between the experimental condition and a control, as the result of the multiplication of two matrices describing behaviors across conditions (the **P **or pattern matrix) and the distribution of genes within those behaviors (the **A **or amplitude matrix). Naturally, the data, **D**, includes noise, so that the full relationship is defined by

Dij≅∑k=1KAikPkj+εij     [1]
 MathType@MTEF@5@5@+=feaafiart1ev1aaatCvAUfKttLearuWrP9MDH5MBPbIqV92AaeXatLxBI9gBaebbnrfifHhDYfgasaacH8akY=wiFfYdH8Gipec8Eeeu0xXdbba9frFj0=OqFfea0dXdd9vqai=hGuQ8kuc9pgc9s8qqaq=dirpe0xb9q8qiLsFr0=vr0=vr0dc8meaabaqaciaacaGaaeqabaqabeGadaaakeaacqWGebardaWgaaWcbaGaemyAaKMaemOAaOgabeaakiabgwKianaaqahabaGaemyqae0aaSbaaSqaaiabdMgaPjabdUgaRbqabaGccqWGqbaudaWgaaWcbaGaem4AaSMaemOAaOgabeaakiabgUcaRGGaciab=v7aLnaaBaaaleaacqWGPbqAcqWGQbGAaeqaaaqaaiabdUgaRjabg2da9iabigdaXaqaaiabdUealbqdcqGHris5aOGaaCzcaiaaxMaacqGGBbWwcqaIXaqmcqGGDbqxaaa@4AD1@

where *D*_*ij *_is the estimated ratio for gene *i *in mutant *j*, *A*_*ik *_is the strength of gene *i *in pattern *k*, *P*_*kj *_is the strength of mutant *j *in pattern *k*, and *ε*_*ij *_is the noise for gene *i *in mutant *j *estimated by the Rosetta error model. BD estimates **A **and **P **by a Markov chain Monte Carlo (MCMC) simulation. For the fixed noise estimate, *ε*, **A **and **P **are inferred from the marginal probability distribution

*p *(**D **| **A,P**) *p *(**A,P**)     [2]

where *p*(**A,P**) is the prior probability and *p *(**D **| **A,P**) is given by the likelihood such that

log⁡p(D|A,P)=−∑i∑j{12εij2(Dij−∑k=1KAikPkj)2}.     [3]
 MathType@MTEF@5@5@+=feaafiart1ev1aaatCvAUfKttLearuWrP9MDH5MBPbIqV92AaeXatLxBI9gBaebbnrfifHhDYfgasaacH8akY=wiFfYdH8Gipec8Eeeu0xXdbba9frFj0=OqFfea0dXdd9vqai=hGuQ8kuc9pgc9s8qqaq=dirpe0xb9q8qiLsFr0=vr0=vr0dc8meaabaqaciaacaGaaeqabaqabeGadaaakeaacyGGSbaBcqGGVbWBcqGGNbWzcqWGWbaCcqGGOaakieqacqWFebarcqGG8baFcqWFbbqqcqGGSaalcqWFqbaucqGGPaqkcqGH9aqpcqGHsisldaaeqbqaamaaqafabaWaaiWaaeaadaWcaaqaaiabigdaXaqaaiabikdaYGGaciab+v7aLnaaDaaaleaacqWGPbqAcqWGQbGAaeaacqaIYaGmaaaaaOWaaeWaaeaacqWGebardaWgaaWcbaGaemyAaKMaemOAaOgabeaakiabgkHiTmaaqahabaGaemyqae0aaSbaaSqaaiabdMgaPjabdUgaRbqabaGccqWGqbaudaWgaaWcbaGaem4AaSMaemOAaOgabeaaaeaacqWGRbWAcqGH9aqpcqaIXaqmaeaacqWGlbWsa0GaeyyeIuoaaOGaayjkaiaawMcaamaaCaaaleqabaGaeGOmaidaaaGccaGL7bGaayzFaaaaleaacqWGQbGAaeqaniabggHiLdaaleaacqWGPbqAaeqaniabggHiLdGccqGGUaGlcaWLjaGaaCzcaiabcUfaBjabiodaZiabc2faDbaa@682F@

The prior is used here to require positivity and to minimize structure in the estimates of **A **and **P **[46].

The analysis with BD is similar mathematically to an analysis with singular value decomposition (SVD) or with principal component analysis (PCA), since all methods estimate two matrices that together reconstruct the data. In both PCA and SVD, orthogonality conditions force each row of **P **to be linearly independent, deriving **P **from either the data matrix (SVD) or the covariance matrix (PCA) using deterministic algorithms. Since patterns of expression related to biological processes will not generally be independent, BD uses the MCMC approach to avoid orthogonality. The resulting rows of **P **are usually easier to relate to biological processes than those from SVD or PCA.

BD was run at each posited dimensionality, *K *(as in equation 1), between 3 and 25, generating a mean estimate and an uncertainty (i.e., standard deviation from samples of the posterior probability distribution) for each element of **A **and **P**. The dimensionality is equivalent to the number of patterns, as the patterns can be viewed as nonorthogonal basis vectors for **D**.

### Persistence and dimensionality

**Figure 4:**
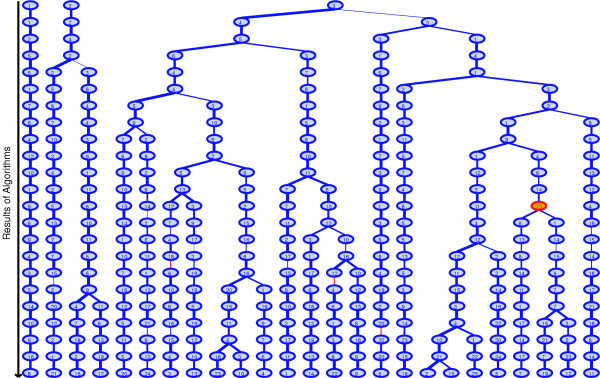
**Relationship of patterns across dimensionalities**. The results for all patterns identified in all runs of Bayesian Decomposition are summarized here. The top row shows three patterns from an analysis with 3 dimensions, while the bottom row shows 25 dimensions. The highlighted node is pattern 13 in 15 dimensions, which is the pattern identified as the mating response. Nodes are connected as described in the text using Pearson correlation measures. The numbers within the nodes are indices and have no intrinsic meaning. Each number provides the row index for **P **and column index for **A **for the analysis at that level.

The results of Bayesian Decomposition across the different estimated dimensionalities, *K*, were compared with ClutrFree in order to visualize stable basis vectors and a persistence measurement on them [49]. Each independent BD analysis provides a tree level, with each pattern represented by a node (see Figure 4). The analysis with the fewest patterns is placed at the top of the tree, and additional levels of analyses are added creating a tree from fewer to larger numbers of patterns. Connections in the tree are created in a greedy way. Level *N+1 *(e.g., 4) is connected to level *N *(e.g., 3) by finding the node in level *N+1 *with the highest correlation to a node in level *N*. The correlation is given by the Pearson correlation for the strength of the assignment of mutants to a pattern (i.e., between *P*_*kj *_for the nodes). These nodes are connected and removed. From the remaining nodes, the maximum correlation between a node at level *N+1 *and one at *N *is found again, and this process is repeated until only a single node remains at level *N+1*. This node is then connected to the node at level *N *that yields the highest correlation.

**Figure 5:**
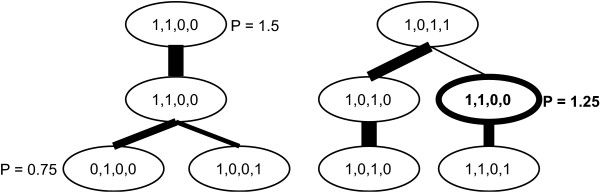
**A sample calculation of the average persistence for a single node**. The average persistence is calculated by comparing the persistence at each node in the tree given in Figure 4. Each assignment of each mutant (4 are shown here) to a pattern is binarized as described in the text, then the average persistence for a node is calculated by checking on the number of times the mutant assigned to the pattern occurs in the connected nodes. The mutant can occur in any branch below the node of interest to be considered as present. If it occurs in multiple child nodes at a single level, that is still treated as a single occurrence for that level. The average for a dimension is then the average of the persistence of all nodes at that level.

Following our previous work [49], we use a measure of persistence to quantify the robustness of a pattern across the variation of the number of patterns. An example calculation is shown in Figure 5. The assignment of a mutant to a pattern (i.e., a node) is binarized based on the mean and uncertainty of the assignment of the mutant to the pattern determined by the MCMC sampling, using a requirement that the mutant be assigned to the pattern at greater than 3*σ *above zero. In Figure 5, it is assumed there are four mutants in a pattern, thus there are four binarized values. Then at each node, each mutant is compared for consistency in presence of the mutant in linked nodes within the tree. For example, for the highlighted node in Figure 5, the first mutant is present in the node above and the node below, so it is present in all 3 connected nodes. For the second mutant it is present in 2, and the third and fourth mutants are absent. The average persistence for the node is therefore (3+2+0+0)/4 = 1.25 as noted in Figure 5. For branches, the mutant status is only required to agree in a single branch to be counted. The average persistence at a dimension is then the average of the persistence for all nodes at that dimension (i.e., a row in Figure 4).

We assessed the dimensionality of the data using measurements of persistence. The persistence was measured for analyses from *3 *– *25 *patterns and the dimension chosen where a significant drop occurred in an otherwise slow monotonic decline, which was expected due to the branching nature of the tree. Figure 2 shows the significant drop between 15 and 16 dimensions, so 15 patterns were chosen for further analysis.

### Ontology and function

To assign ontological terms to the genes contained in basis vectors, we annotated our data using the gene ontologies from the Comprehensive Yeast Genome Database (CYGD) hosted at the Munich Information center for Protein Sequences (MIPS) [51,72]. The analysis here utilizes the Functional Catalog (FunCat) format that describes each gene using a hierarchical ontological model.

Similar to persistence, we defined enhancement as a measure of the over-representation, or under representation, of a gene function in a subset of the data [47]. It is the ratio of the frequency of occurrence of genes annotated by a particular ontological term in the pattern to the frequency of occurrence of the same term in the whole dataset,

e(t,p)=gp/npG/N,     [4]
 MathType@MTEF@5@5@+=feaafiart1ev1aaatCvAUfKttLearuWrP9MDH5MBPbIqV92AaeXatLxBI9gBaebbnrfifHhDYfgasaacH8akY=wiFfYdH8Gipec8Eeeu0xXdbba9frFj0=OqFfea0dXdd9vqai=hGuQ8kuc9pgc9s8qqaq=dirpe0xb9q8qiLsFr0=vr0=vr0dc8meaabaqaciaacaGaaeqabaqabeGadaaakeaacqWGLbqzcqGGOaakcqWG0baDcqGGSaalcqWGWbaCcqGGPaqkcqGH9aqpdaWcaaqaaiabdEgaNnaaBaaaleaacqWGWbaCaeqaaOGaei4la8IaemOBa42aaSbaaSqaaiabdchaWbqabaaakeaacqWGhbWrcqGGVaWlcqWGobGtaaGaeiilaWIaaCzcaiaaxMaacqGGBbWwcqaI0aancqGGDbqxaaa@441D@

with *g*_*p *_being the number of genes annotated with the term *t *in pattern *p*, *n*_*p *_the total number of genes in the pattern *p*, *G *the number of genes annotated by the term *t *in the data, and *N *being the total number of genes in the dataset. In addition, we apply a hypergeometric test to estimate a p-value for each term. Function was then determined by inspection of enhanced ontological terms.

### Transcription factor analysis

The genes were also analyzed for the patterns determined to be related to signaling pathways by exploration of the ten genes most strongly associated with the pattern. Each gene was analyzed using the Saccharomyces Genome Database [73] to determine whether it was known to be associated with mating or filamentation processes and to determine if it was directly regulated by the Ste12p transcription factor.

## Authors' contributions

GB performed analysis with BD and ClutrFree, including identification of biological processes from gene ontology measurements. KS and JMC provided advice and guidance on development of ClutrFree for these analyses. MFO oversaw the project and did gene ontology and transcription factor analysis on patterns 13 and 15.
